# Physical-Layer Security, Quantum Key Distribution, and Post-Quantum Cryptography

**DOI:** 10.3390/e24070935

**Published:** 2022-07-06

**Authors:** Ivan B. Djordjevic

**Affiliations:** Department of Electrical and Computer Engineering, University of Arizona, 1230 E. Speedway Blvd., Tucson, AZ 85721, USA; ivan@email.arizona.edu; Tel.: +1-520-626-5119

The growth of data-driven technologies, 5G, and the Internet pose enormous pressure on underlying information infrastructure. There are numerous proposals on how to deal with the possible capacity crunch [1]. However, the security of both optical and wireless networks lags behind reliable and spectrally efficient transmission [2]. Significant achievements have been recently made in the arenas of quantum computing [3] and quantum communication [4,5]. Because most conventional cryptography systems rely on computational security, which guarantees security against an efficient eavesdropper for a limited time, with advancements in quantum computing, this security can be compromised. To solve for these problems, various schemes providing the perfect/unconditional security have been proposed, including physical-layer security (PLS), quantum key distribution (QKD), and post-quantum cryptography. Unfortunately, it is still unclear how to integrate those different proposals with higher-level cryptography schemes. Thus, the purpose of this Special Issue was to integrate these various approaches and enable the next generation of cryptography systems whose security cannot be broken by quantum computers.

The topics addressed in this Special Issue include physical-layer security [2], quantum key distribution (QKD) [2], post-quantum cryptography [6], quantum-enhanced cryptography [7], stealth communication [2], and covert communication [8]. There are 14 papers published in this Special Issue, distributed as follows: 1 review paper, 1 perspective paper, and 12 articles.

In the review paper [9], authors apply the restricted Eve’s concept to the satellite-to-satellite secret key distillation. In conventional QKD, it is assumed that Eve is the omnipotent, limited only by the laws of physics. This represents an unreasonable assumption for certain applications, where the presence of Eve is easy to detect, such as free-space optical communications, particularly satellite-to-satellite communications. By introducing geometrical optics within a restricted model, authors have shown that the secret key rate (SKR) can be significantly improved compared to the conventional QKD. Authors analyze SKRs from Bob’s perspective through the exclusion zone approach and from Eve’s perspective through dynamic positioning of the receiver aperture.

In the perspective paper [10], the author discusses how to build a global quantum communication network (QCN) by interconnecting the disconnected terrestrial QCNs through LEO satellite QCN, based on the cluster state concept. This heterogenous global QCN will provide unprecedented security for future 5G+/6G wireless networks, Internet of Things (IoT), optical networks, and autonomous vehicles.

In the first article paper [11], authors discuss the underwater QKD. Authors apply measurement-device-independent (MDI) QKD with the zero-photon catalysis (ZPC) performed at the emitter of one side to improve the SKR and extend the transmission distance. Numerical results indicate that the proposed ZPC-based scheme outperforms the corresponding single photon subtraction-based scheme in the extreme asymmetric case.

In the second article paper [12], the author describes how to build the multipartite QCN based on the surface code (SC) concept. The key idea is to simultaneously entangle multiple nodes in an arbitrary topology based on the SC approach. The author also describes how to extend the transmission distance between nodes to beyond 1000 km using SCs.

In the third article paper [13], authors introduce an open-destination MDI QKD network that provides security against untrusted relays and all detector side-channel attacks, in which all user users are capable of distributing keys with the help of other users.

In the fourth article paper [14], authors introduce a QKD protocol which employs the mean multi-king problem in which a sender shares a bit sequence with receivers as a secret key. Authors study the relation between eavesdropper’s information gain and disturbance introduced into legitimate users’ information, known as the information disturbance theorem, used for the BB84 protocol. Authors show that Eve’s extracting information disturbs the quantum states and increases the error probability, as expected.

In the fifth article paper [15], authors introduce a QKD post-processing method, cubically raising the SKR in the number of double matching detection events. In the proposed protocol, contrary to the conventional QKD protocols, the secret bits rely on Bob’s measurement basis selection rather than Alice’s transmitted bits. Furthermore, the proposed protocol combines the sifting, reconciliation, and amplification into a unique process, thus requiring a single-round iteration without sending redundancy bits.

In the sixth article [16], authors study a recent proposal for quantum identity authentication from Zawadzki [17] and formally prove that the corresponding protocol is insecure.

In the seventh article [18], authors study the phase-matching QKD (PM-QKD) protocol, employing discrete-phase randomization and the post-compensation phase to quadratically improve the SKR. Unfortunately, according to the authors, the discrete-phase randomization opens a security loophole. Authors introduce the unambiguous state discrimination measurement and the photon-number-splitting attack against PM-QKD with imperfect phase randomization, demonstrating the rigorous security of decoy state PM-QKD with a discrete-phase randomization protocol.

In the eight article [19], authors introduce a nonclassical attack on the QKD system and propose a corresponding countermeasure method. The proposed attack is based on the sync pulses attenuated to a photon level to determine the signaling interval. To solve this attack, authors propose using variable power synchronizing pulses at varying lengths, combined with the controlled signal attenuation.

In the nineth article paper [20], an entanglement-based QKD protocol is proposed that employs a modified symmetric version of the Bernstein–Vazirani algorithm to achieve secure and efficient key distribution, with two variants presented (fully symmetric and semi-symmetric).

In the 10th article paper [21], related to the physical-layer security, authors study the impact of injection and jamming attacks during the advantage distillation in a MIMO wireless system and show that the man-in-the-middle attack can be mounted as long as the attacker has one extra antenna with respect to the legitimate users. To solve for this problem, authors propose reducing the injection attack by using a particularly designed pilot randomization technique. Then, by employing a game-theoretic approach, authors evaluate the optimal strategies available to the legitimate users in the presence of reactive jammers.

In the 11th article [22], authors introduce a Bayesian probabilistic algorithm that incorporates all published information in a qubit-based synchronization protocol to efficiently determine the clock offset without sacrificing any secure key. Given that the output of the algorithm is a probability, it can be used to quantify the synchronization confidence.

In the final article paper [23], related to the secure computation, authors present randomized versions of two known oblivious transfer protocols—one being quantum and the other being post-quantum with ring learning and an error assumption, thus demonstrating their security in the quantum universal composability framework with the use of a common reference string model.
